# Supplementary material for: Effect of INTELLiVENT-ASV versus Conventional Ventilation on Ventilation Intensity in Patients with COVID-19 ARDS—An Observational Study
Source: J Clin Med. 2021 Nov 19;10(22):5409. doi: 10.3390/jcm10225409 (PMC8622732; doi:10.3390/jcm10225409)
Supplement: Supplementary file 1 [file jcm-10-05409-s001.zip › jcm-1433773-supplementary.pdf]

Supplement with

Effect of INTELLiVENT-ASV versus Conventional Ventilation on Ventilation Intensity in Patients with COVID-19 ARDS—an observational study

Laura A. Buiteman-Kruizinga, Hassan E. Mkadmi, Ary Serpa Neto, Matthijs D. Kruizinga, Michela Botta, Marcus J. Schultz, Frederique Paulus and Pim L.J. van der Heiden

Figure S1.. 2
Figure S2.. 3
Figure S3.. 4
Figure S4.. 5
Figure S5.. 7
Figure S6.. 8
Figure S7.. 9
Table S1a-h.. 10

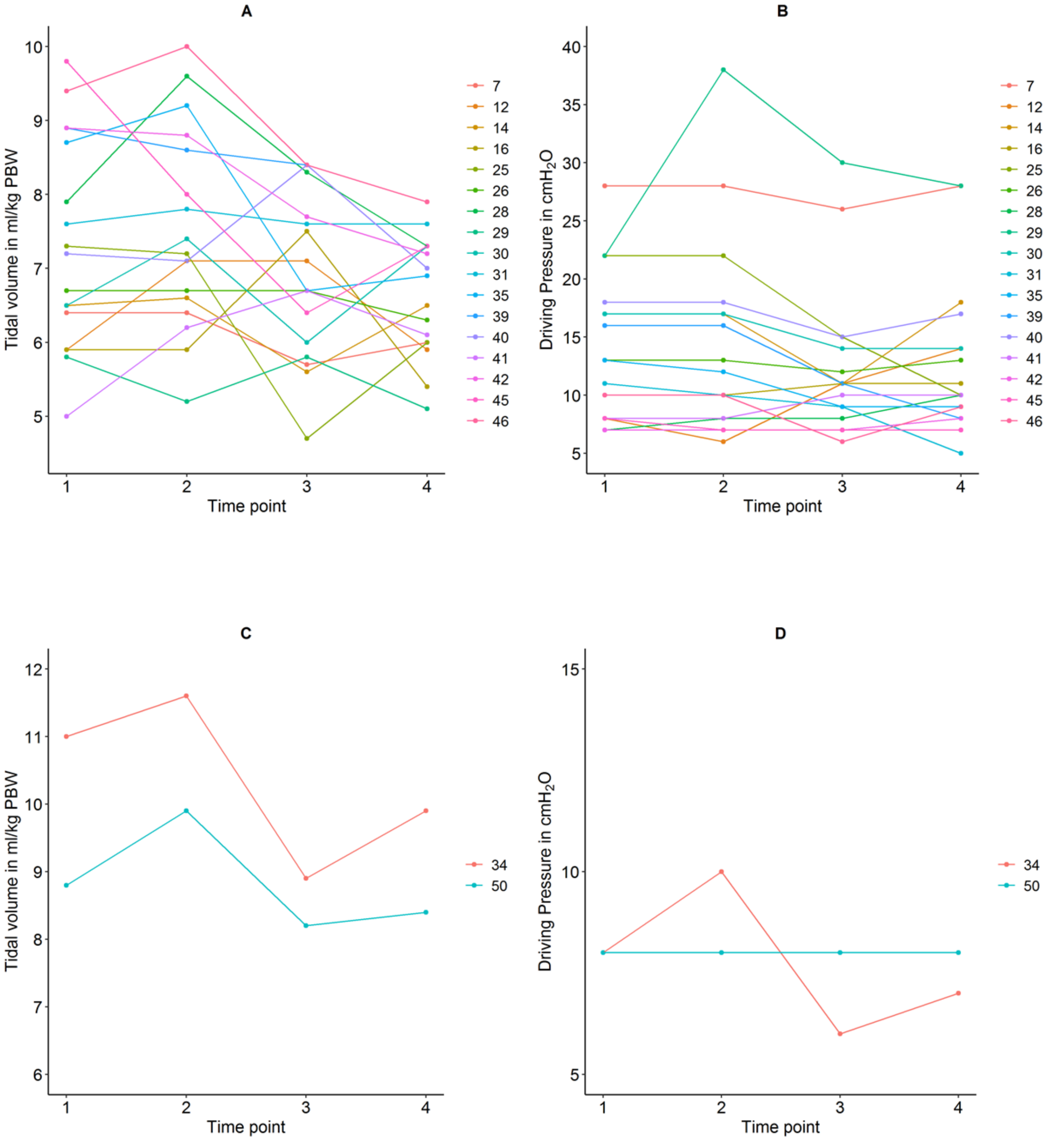

Figure S1. Plot of a subset of patients of individual changes in V_T and ΔP. Plot of a subset of patients with a decrease in V_T with INTELLiVENT-ASV, as determined 1 hour before conversion and 2 hours after conversion with (a) a decrease to a V_T below 8 ml/kg PBW; (b) the effect on ΔP in the same patients; (c) a decrease to a V_T above 8ml/kg PBW; and (d) the effect on ΔP in the same patients. Timepoints 1 and 2 are with conventional ventilation, and 3 and 4 are with INTELLiVENT-ASV.

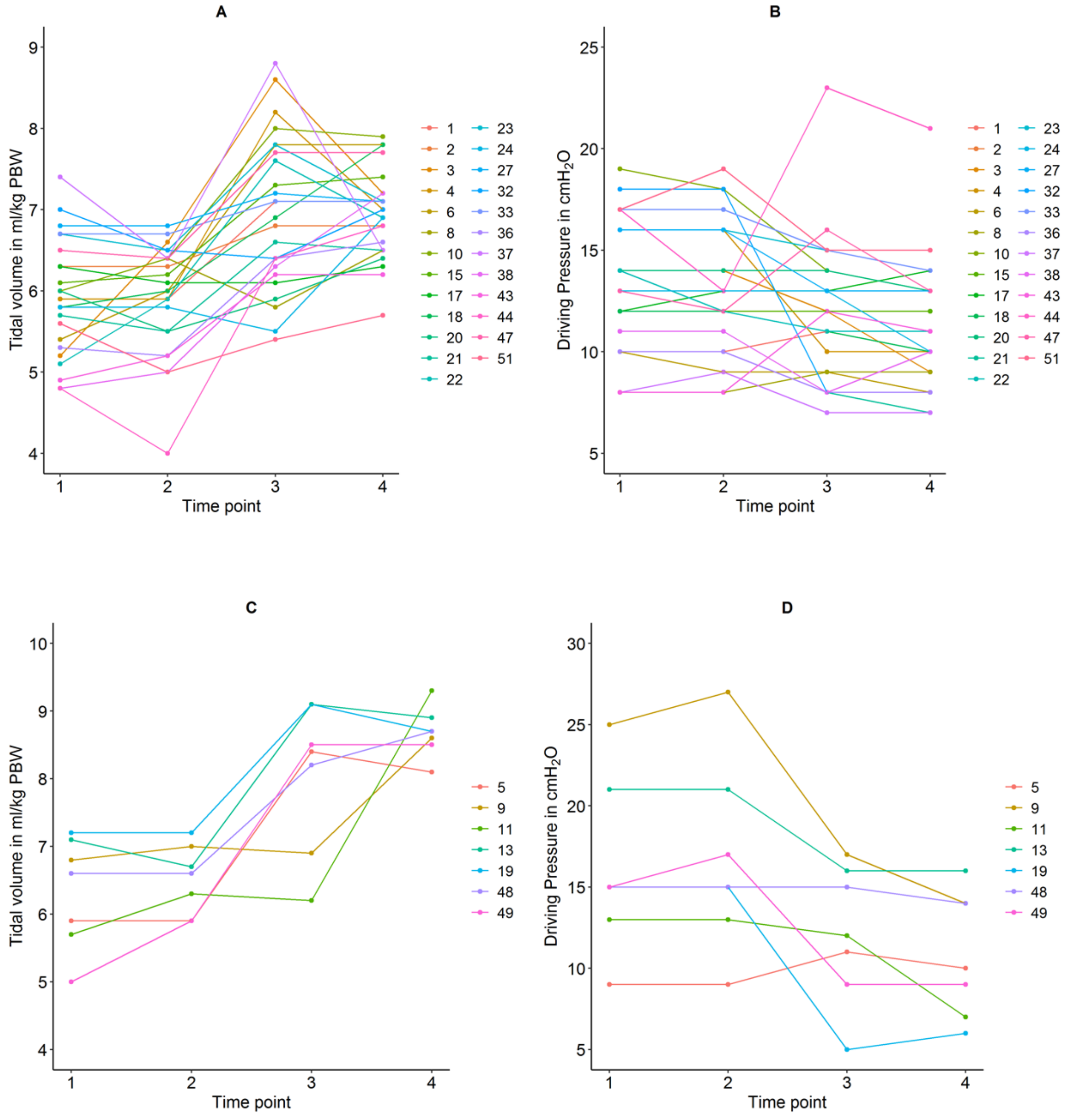

Figure S2. Plot of a subset of patients of individual changes in V_T and ΔP. Plot of a subset of patients with an increase in V_T with INTELLiVENT-ASV, as determined 1 hour before conversion and 2 hours after conversion with (a) an increase to a V_T below 8 ml/kg PBW; (b) the effect on ΔP in the same patients; (c) an increase to a V_T above 8ml/kg PBW; and (d) the effect on ΔP in the same patients. Timepoints 1 and 2 are with conventional ventilation, and 3 and 4 are with INTELLiV-ENT-ASV.

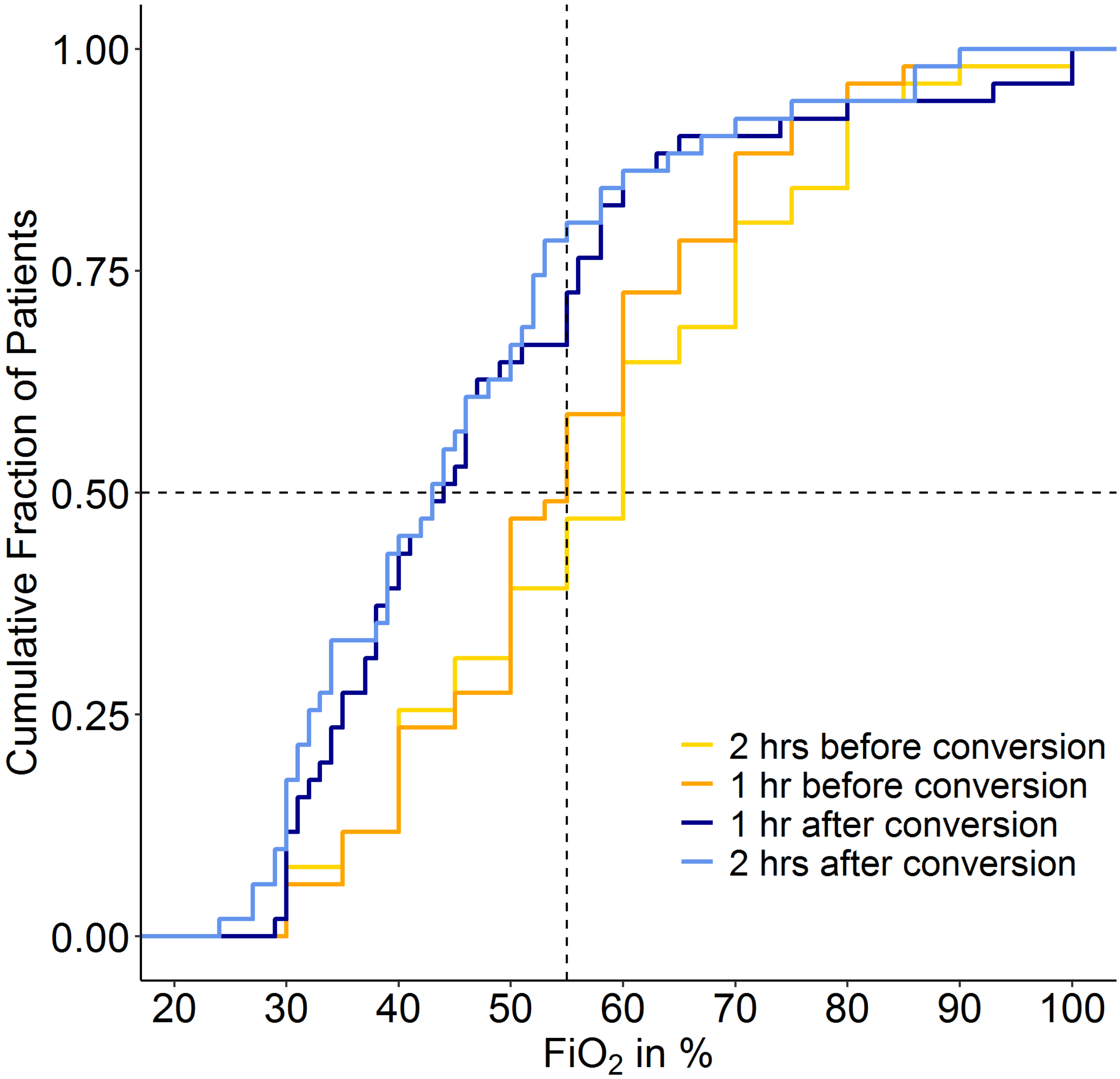

Figure S3. Cumulative frequency distribution of minute volume in liters. The plot shows minute volume from before to after the conversion between conventional ventilation and INTELLiVENT-ASV. Vertical dotted lines represent the median at the last hour before the conversion, and horizontal dotted lines show the respective proportion of patients reaching each cutoff.

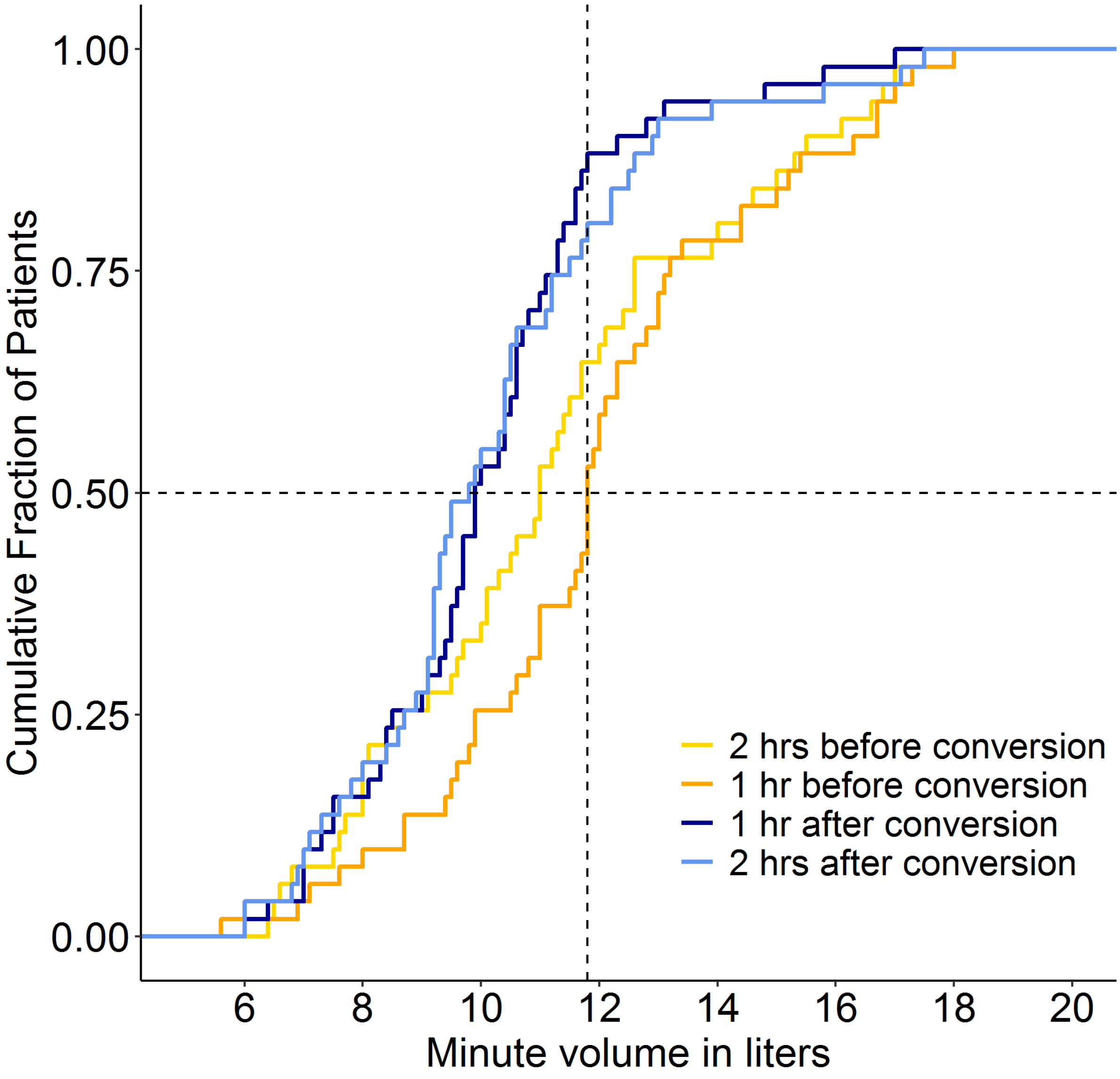

Figure S4. Cumulative frequency distribution of the fraction of inspired oxygen. The plot shows the FiO_2 from before to after the conversion between conventional ventilation and INTELLiVENT-ASV. Vertical dotted lines represent the median at the last hour before the conversion, and horizontal dotted lines show the respective proportion of patients reaching each cutoff.

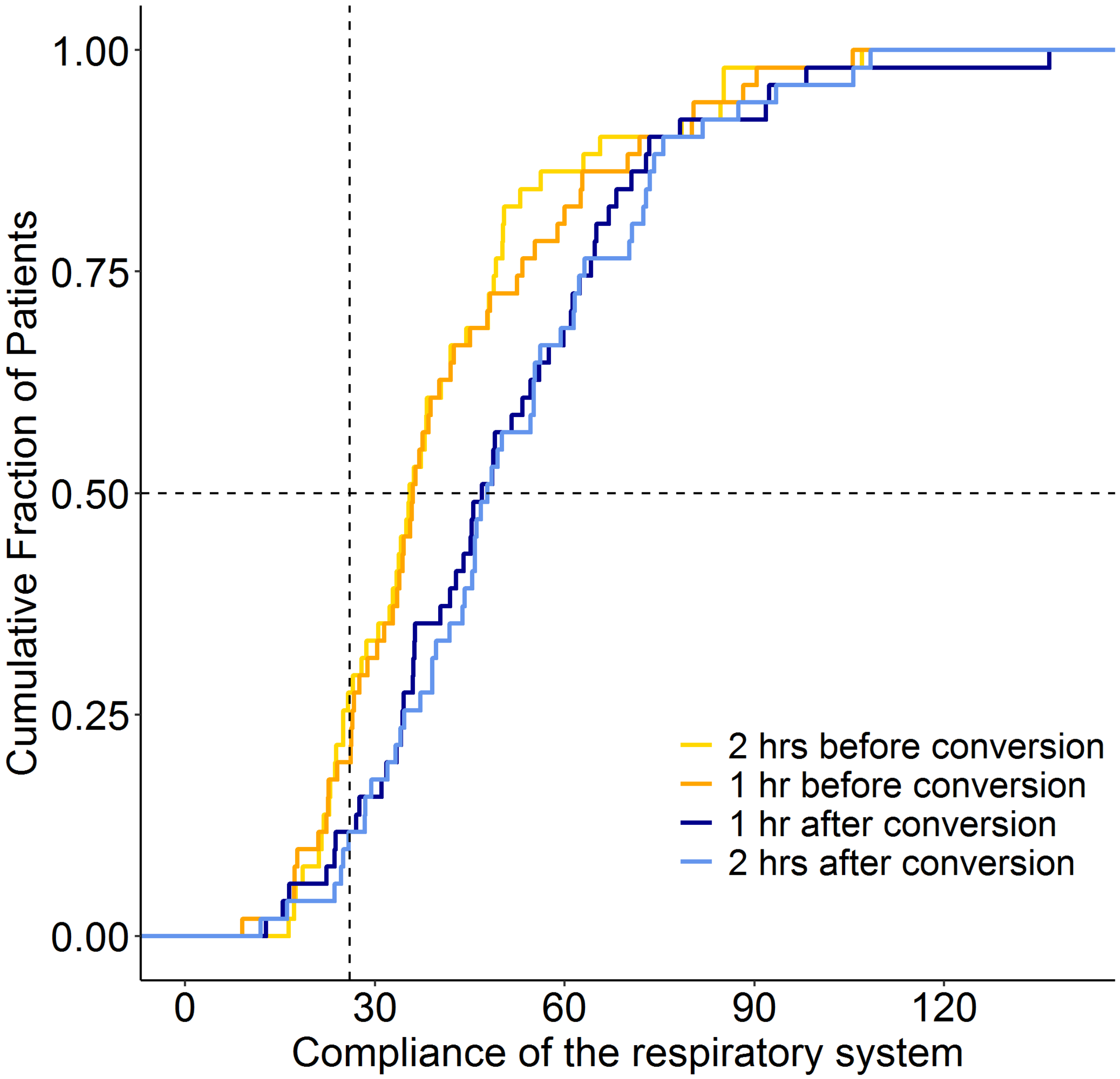

Figure S5. Cumulative frequency distribution of the compliance of the respiratory system in mL/cm H_2O liters. The plot shows the C_{RS} from before to after the conversion between conventional ventilation and INTELLiVENT-ASV. Vertical dotted lines represent the median at the last hour before the conversion, and horizontal dotted lines show the respective proportion of patients reaching each cutoff.

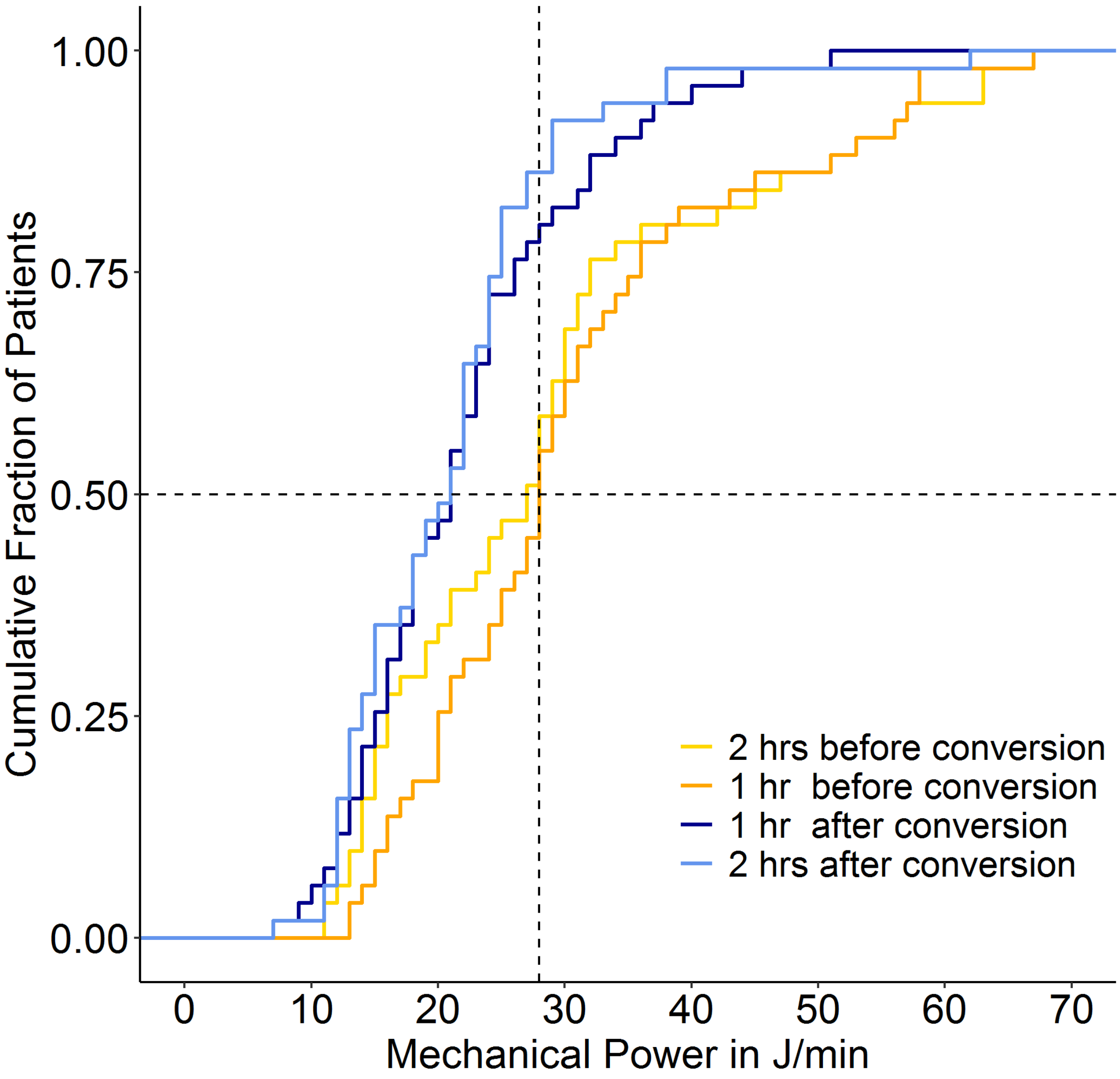

Figure S6. Cumulative frequency distribution of MP with an alternate equation. The plot shows MP from before to after the conversion between conventional ventilation and INTELLiVENT-ASV. Vertical dotted lines represent the median at the last hour before the conversion, and horizontal dotted lines show the respective proportion of patients reaching each cutoff. MP was calculated as 0·098 * RR * V_T *(Pinsp + PEEP).

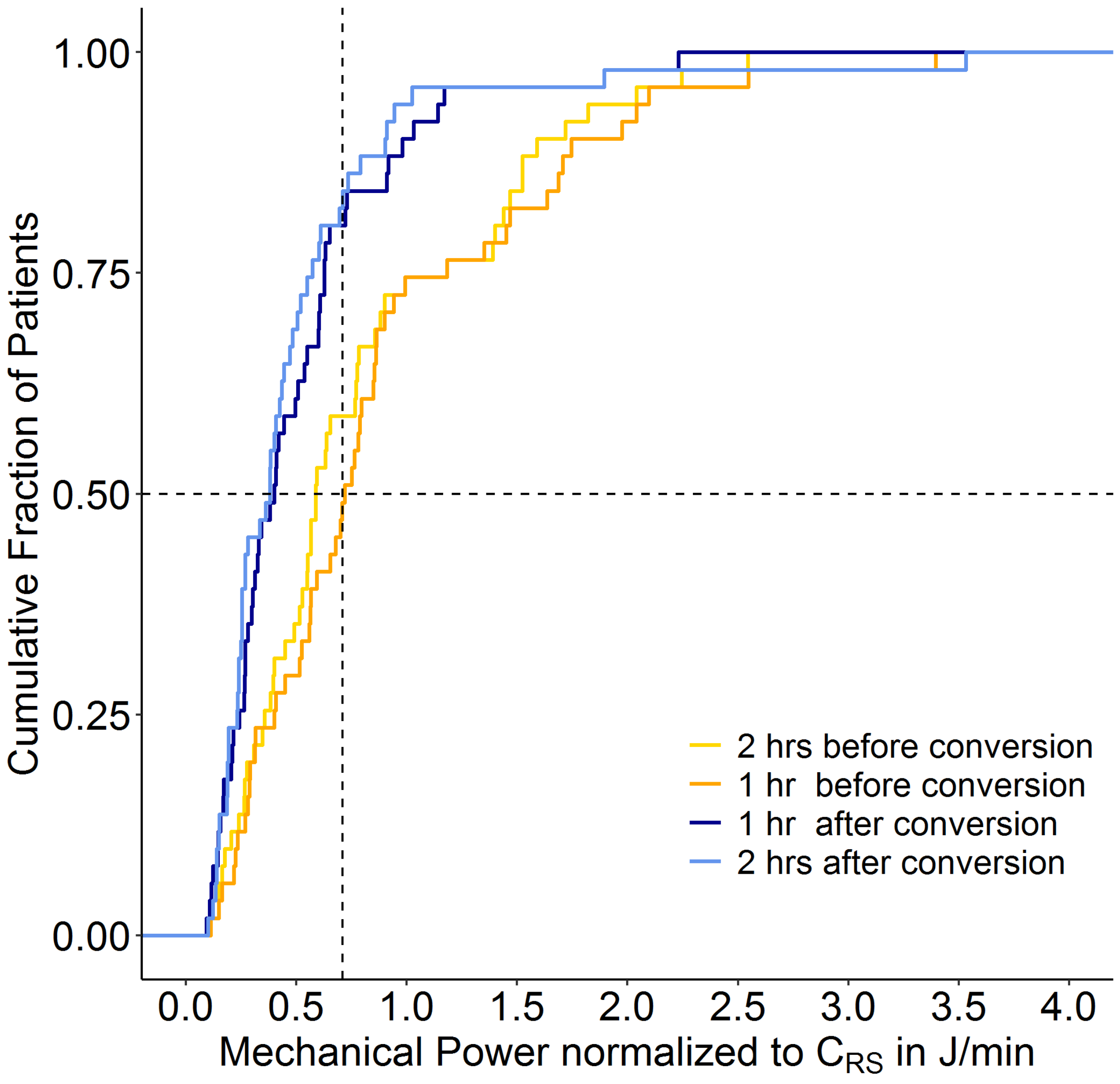

Figure S7. Cumulative frequency distribution of MP normalized to C_{RS}. The plot shows MP from before to after the conversion between conventional ventilation and INTELLiVENT-ASV. Vertical dotted lines represent the median at the last hour before the conversion, and horizontal dotted lines show the respective proportion of patients reaching each cutoff. C_{RS} (mL/cm H_2O) = $V_T/\Delta P$, and MP_{NORM} (J/min per mL/cm H_2O) = MP/C_{RS}.

Table S1a. Individual ventilation data of tidal volume.

Study ID	Tidalvolume time point 1	Tidalvolume time point 2	Tidalvolume time point 3	Tidalvolume time point 4
1	420	420	501	504
2	504	500	551	554
3	468	595	777	652
4	400	420	560	478
5	400	405	568	553
6	420	430	561	562
7	481	483	427	452
8	450	480	438	491
9	464	479	468	585
10	450	480	600	596
11	397	440	435	654
12	402	482	484	398
13	501	476	646	635
14	600	604	516	598
15	459	466	544	553
16	530	533	671	514
17	485	473	468	485
18	420	430	496	561
19	543	540	683	650
20	477	441	468	508
21	502	482	583	573
22	360	412	534	482
23	350	337	405	370
24	420	427	403	501
25	360	353	231	294
26	439	436	436	416
27	424	423	444	442
28	596	723	626	551
29	384	342	383	334
30	359	409	331	399
31	537	589	538	528
32	591	547	536	588
33	487	490	515	520
34	681	718	551	612
35	624	663	480	496
36	381	375	460	475
37	383	333	455	339
38	417	423	545	623
39	567	552	539	505
40	413	402	477	401
41	403	502	546	494
42	749	739	646	605
43	393	420	503	499
44	387	294	513	543
45	677	561	449	510
46	656	700	589	553
47	484	482	577	574
48	418	413	518	547
49	375	445	635	636
50	628	706	587	593
51	367	328	357	375

Table S1b. Individual ventilation data of PEEP.

Study ID	PEEP time point 1	PEEP time point 2	PEEP time point 3	PEEP time point 4
1	14	14	14	14
2	17	17	16	16
3	8	8	10	10
4	10	10	7	5
5	20	20	16	17
6	10	12	12	12
7	8	8	8	8
8	8	9	9	10
9	8	6	6	7
10	16	16	12	12
11	8	8	8	8
12	8	8	8	9
13	5	5	8	8
14	8	10	14	8
15	10	10	8	8
16	12	12	12	12
17	16	14	13	12
18	10	12	14	12
19	10	10	12	12
20	16	16	14	14
21	12	12	12	11
22	14	16	16	16
23	8	10	11	11
24	9	12	12	11
25	10	10	12	12
26	12	12	12	11
27	12	12	12	12
28	10	10	12	11
29	7	5	8	7
30	7	7	10	9
31	8	8	9	11
32	10	10	10	10
33	12	12	10	10
34	12	10	10	12
35	5	5	7	7
36	10	8	10	10
37	11	12	13	13
38	10	10	10	10
39	10	10	9	10
40	15	15	15	12
41	14	14	15	15
42	8	8	8	8
43	10	10	10	6
44	15	12	11	11
45	12	14	11	12
46	10	10	10	10
47	10	10	10	10
48	10	10	10	8
49	5	5	12	12
50	8	8	10	10
51	12	10	10	10

Table S1c. Individual ventilation data of Pmax.

Study ID	Pmax time point 1	Pmax time point 2	Pmax time point 3	Pmax time point 4
1	31	32	26	25
2	38	37	31	31
3	26	24	23	19
4	29	32	18	15
5	38	38	32	32
6	25	30	28	27
7	43	43	38	47
8	17	21	18	19
9	43	41	33	28
10	43	42	32	33
11	22	22	20	15
12	17	15	20	26
13	40	37	33	32
14	29	29	27	25
15	23	23	23	23
16	27	25	26	24
17	33	31	28	27
18	26	30	29	28
19	30	30	17	18
20	41	48	39	36
21	26	26	21	18
22	30	30	29	29
23	25	28	30	28
24	22	29	29	22
25	34	35	29	34
26	27	27	26	26
27	35	40	32	32
28	18	19	21	22
29	34	43	38	37
30	25	26	24	23
31	20	18	17	16
32	35	35	27	24
33	31	31	28	28
34	20	20	16	19
35	18	17	16	16
36	23	24	20	21
37	19	21	20	20
38	23	23	21	24
39	26	30	20	18
40	43	43	37	30
41	22	22	29	28
42	15	15	15	16
43	23	23	22	17
44	39	33	42	40
45	20	21	18	19
46	20	20	16	19
47	28	29	26	23
48	27	26	26	22
49	20	22	21	21
50	20	20	18	18
51	35	36	27	28

Table S1d Individual ventilation data of Pplat.

Study ID	Pplat time point 1	Pplat time point 2	Pplat time point 3	Pplat time point 4
1	26	26	24	24
2	25	25	25	25
3	23	22	22	19
4	26	26	17	15
5	29	29	27	27
6	20	21	21	20
7	36	36	34	36
8	16	17	18	19
9	33	33	23	21
10	35	34	26	25
11	21	21	20	15
12	16	14	19	23
13	26	26	24	24
14	25	25	25	25
15	22	22	20	20
16	22	22	23	23
17	28	27	26	26
18	22	24	25	22
19	25	25	17	18
20	30	30	28	27
21	22	22	20	18
22	28	28	27	27
23	24	26	26	25
24	22	25	25	21
25	32	32	27	31
26	25	25	24	24
27	28	28	25	25
28	17	18	20	21
29	29	43	38	35
30	24	24	24	24
31	19	18	17	16
32	28	28	18	18
33	29	29	25	24
34	20	20	16	19
35	18	17	16	16
36	20	18	18	18
37	19	21	20	20
38	21	21	18	20
39	26	26	20	18
40	33	33	30	29
41	22	22	25	25
42	15	15	15	16
43	18	18	22	17
44	32	25	34	32
45	20	21	18	19
46	20	20	16	19
47	23	22	26	23
48	25	26	25	22
49	20	22	21	21
50	16	16	18	18
51	29	29	25	25

Table S1e. Individual ventilation data of the respiratory rate.

Study ID	RR time point 1	RR time point 2	RR time point 3	RR time point 4
1	30	30	19	19
2	33	33	19	19
3	30	30	17	21
4	16	18	21	19
5	40	40	21	16
6	25	25	15	14
7	32	32	35	39
8	16	20	14	15
9	23	24	24	15
10	36	36	21	21
11	30	30	17	17
12	20	21	19	18
13	30	30	16	16
14	25	22	27	26
15	20	20	19	19
16	16	16	12	12
17	20	25	20	20
18	25	25	20	18
19	30	30	15	21
20	30	30	26	24
21	25	25	11	15
22	20	30	16	19
23	22	30	22	23
24	20	30	22	20
25	28	28	37	32
26	25	25	21	23
27	36	34	28	29
28	24	19	18	23
29	29	38	28	30
30	28	27	26	23
31	17	17	14	15
32	32	32	18	17
33	24	24	20	18
34	14	19	12	13
35	18	18	12	12
36	31	30	17	19
37	26	36	18	21
38	30	30	20	15
39	20	25	21	23
40	34	34	15	17
41	27	22	17	16
42	14	14	18	16
43	30	30	16	15
44	30	40	15	13
45	12	24	17	13
46	10	11	15	15
47	13	15	19	18
48	27	28	15	12
49	30	32	18	16
50	22	23	19	21
51	36	40	32	34

Table S1f. Individual ventilation data of tidal volume in ml/kg PBW.

Stud IDy	Tidalvolume_mlkg time point 1	Tidalvolume_mlkg time point 2	Tidalvolume_mlkg time point 3	Tidalvolume_mlkg time point 4
1	5,9	5,9	7,10	7,1
2	6,3	6,3	6,80	6,8
3	5,2	6,6	8,60	7,2
4	5,9	5,9	8,20	7,0
5	5,9	5,9	8,40	8,1
6	5,4	6,0	7,80	7,8
7	6,4	6,4	5,70	6,0
8	6,0	6,4	5,80	6,5
9	6,8	7,0	6,90	8,6
10	6,0	6,4	8,00	7,9
11	5,7	6,3	6,20	9,3
12	5,9	7,1	7,10	5,9
13	7,1	6,7	9,10	8,9
14	6,5	6,6	5,60	6,5
15	6,1	6,2	7,30	7,4
16	5,9	5,9	7,50	5,4
17	6,3	6,1	6,10	6,3
18	5,8	6,0	6,90	7,8
19	7,2	7,2	9,10	8,7
20	6,0	5,5	5,90	6,4
21	5,7	5,5	6,60	6,5
22	5,1	5,9	7,60	6,9
23	6,7	6,5	7,80	7,1
24	5,8	5,8	5,50	6,9
25	7,3	7,2	4,70	6,0
26	6,7	6,7	6,70	6,3
27	6,8	6,8	7,20	7,1
28	7,9	9,6	8,30	7,3
29	5,8	5,2	5,80	5,1
30	6,5	7,4	6,00	7,3
31	7,6	7,8	7,60	7,6
32	7,0	6,5	6,40	7,0
33	6,7	6,7	7,10	7,1
34	11,0	11,6	8,90	9,9
35	8,7	9,2	6,70	6,9
36	5,3	5,2	6,40	6,6
37	7,4	6,4	8,80	6,5
38	4,8	5,0	6,30	7,2
39	8,9	8,6	8,40	7,9
40	7,2	7,1	8,40	7,0
41	5,0	6,2	6,70	6,1
42	8,9	8,8	7,70	7,2
43	4,9	5,2	6,20	6,2
44	4,8	4,0	6,40	6,8
45	9,8	8,0	6,40	7,3
46	9,4	10,0	8,40	7,9
47	6,5	6,4	7,70	7,7
48	6,6	6,6	8,20	8,7
49	5,0	5,9	8,50	8,5
50	8,8	9,9	8,20	8,4
51	5,6	5,0	5,40	5,7

Table S1g. Individual ventilation data of the P/F ratio at the baseline.

Study ID	PF ratio
1	108
2	157
3	167
4	89
5	125
6	85
7	141
8	74
9	333
10	140
11	215
12	121
13	198
14	95
15	158
16	165
17	126
18	98
19	165
20	98
21	135
22	150
23	112
24	93
25	168
26	227
27	138
28	94
29	107
30	139
31	68
32	101
33	94
34	65
35	221
36	129
37	203
38	113
39	88
40	100
41	92
42	230
43	189
44	104
45	83
46	85
47	137
48	125
49	231
50	118
51	95

Table S1h. Individual ventilation data of calculated compliance of the respiratory system.

Study ID	C_{RS} time point 1	C_{RS} time point 2	C_{RS} time point 3	C_{RS} time point 4
1	42,00	42,00	45,55	45,82
2	63,00	62,50	61,22	61,56
3	33,43	42,50	64,75	72,44
4	25,00	26,25	56,00	47,80
5	44,44	45,00	51,64	55,30
6	42,00	47,78	62,33	70,25
7	17,18	17,25	16,42	16,14
8	56,25	60,00	48,67	54,56
9	18,56	17,74	27,53	41,79
10	23,68	26,67	42,86	45,85
11	30,54	33,85	36,25	93,43
12	50,25	80,33	44,00	28,43
13	23,86	22,67	40,38	39,69
14	35,29	35,53	46,91	33,22
15	38,25	38,83	45,33	46,08
16	53,00	53,30	61,00	46,73
17	40,42	36,38	36,00	34,64
18	35,00	35,83	45,09	56,10
19	36,20	36,00	136,60	108,33
20	34,07	31,50	33,43	39,08
21	50,20	48,20	72,88	81,86
22	25,71	34,33	48,55	43,82
23	21,88	21,06	27,00	24,67
24	32,31	32,85	31,00	50,10
25	16,36	16,05	15,40	29,40
26	33,77	33,54	36,33	32,00
27	26,50	26,44	34,15	34,00
28	85,14	90,38	78,25	55,10
29	17,45	9,00	12,77	11,93
30	21,12	24,06	23,64	28,50
31	48,82	58,90	59,78	105,60
32	32,83	30,39	67,00	73,50
33	28,65	28,82	34,33	37,14
34	85,13	71,80	91,83	87,43
35	48,00	55,25	53,33	55,11
36	38,10	37,50	57,50	59,38
37	47,88	37,00	65,00	48,43
38	37,91	38,45	68,13	62,30
39	35,44	34,50	49,00	63,13
40	22,94	22,33	31,80	23,59
41	50,38	62,75	54,60	49,40
42	107,00	105,57	92,29	75,63
43	49,13	52,50	41,92	45,36
44	22,76	22,62	22,30	25,86
45	84,63	80,14	64,14	72,86
46	65,60	70,00	98,17	61,44
47	37,23	40,17	36,06	44,15
48	27,87	27,53	34,53	39,07
49	25,00	26,18	70,56	70,67
50	78,50	88,25	73,38	74,13
51	21,59	17,26	23,80	25,00
